# Opinion on “Current status of MSK radiology training: an international survey by the European Society of Musculoskeletal Radiology (ESSR) Young Club”

**DOI:** 10.1186/s13244-021-01148-6

**Published:** 2022-01-28

**Authors:** Alejandra Duarte, Rodrigo Borrero Leon

**Affiliations:** 1 Head of Musculoskeletal Section of Radiology Department, Fundacion Santa Fe de Bogotá, Carrera 7 # 117-15, Bogota, Colombia; 2 Radiology Resident, Fundación Santa Fe de Bogotá – Universidad El Bosque, Avenida Carrera 7 # 117-15, Bogotá, Colombia

In the article written by Albano et al. titled “Current status of MSK radiology training: an international survey by the European Society of Musculoskeletal Radiology (ESSR) Young Club” [1], the authors talk about training programs around the world, specifically on the musculoskeletal (MSK) field expressing their concern about education in this area worldwide. To have an initial approximation of the current status of MSK radiology training, the researchers made a 20-question survey and exposed the perception radiology residents have about their experience and exposure to MSK cases, academic reviews, meetings, and research.

Patients referred by specialties such as orthopedic surgery, internal medicine, family practice, emergency medicine, and rheumatology [2] for radiological MSK examinations have increased exponentially, and general radiologists must be well prepared to understand and correctly interpret the examinations as well as develop research skills, especially in academic centers.

We agree the survey had very few participants (only 86 countries included with 972 single surveys). For instance, our country Colombia only had 7 single surveys and now we have 18 radiology residency programs and 350 residents approximately.

Our residency program is one of the most demanding both academically and professionally in our country, with over 4,875 MRIs of the joints so far this year and 1–2 MSK radiologist dedicated to MSK MRI exclusively per day. Our residents have 2-month rotations during their radiology training with great exposure to MSK MRI. This is just an example of a residency program in South America, and like the authors we are also concerned about the quality in MSK training in young radiologists worldwide. A paper published by ACR on 2007 [3] proposed that a radiology Residency program that satisfies ACGME´s requirements should include at least three 4-week rotations, which is far from what the survey showed and far from our reality.

Giving the extraordinary effort of the ESR and ESSR to improve MSK radiology training among young radiologist, we consider the effort should be expanded to older radiologists who in their daily practice are confronted to challenging MSK cases. Alliances with radiology societies could improve MSK training, for instance, implementing EDiMSK in countries like Colombia where there are no official MSK fellowship programs. Additionally, EDiMSK could also be accepted as an official postgraduate qualification as in some European Countries [4].

## Data Availability

Not applicable.
